# Dual effects of TGF-β on ERα-mediated estrogenic transcriptional activity in breast cancer

**DOI:** 10.1186/1476-4598-8-111

**Published:** 2009-11-27

**Authors:** Yongsheng Ren, Liyu Wu, Andra R Frost, William Grizzle, Xu Cao, Mei Wan

**Affiliations:** 1 Department of Pathology, University of Alabama at Birmingham, Birmingham, Alabama 35249 USA

## Abstract

**Background:**

TGF-β resistance often develops in breast cancer cells that in turn overproduce this cytokine to create a local immunosuppressive environment that fosters tumor growth and exacerbates the invasive and metastatic behavior of the tumor cells themselves. Smads-mediated cross-talk with the estrogen receptor has been implied to play an important role in development and/or progression of breast cancer. We investigated how TGF-β regulates ERα-induced gene transcription and potential mechanisms of frequent TGF-β resistance in breast cancer.

**Methods:**

Effect of TGF-β on ERα-mediated gene transcription was investigated in breast cancer cell lines using transient transfection, real-time PCR, sequential DNA precipitation, and small interfering RNA assays. The expression of Smads on both human breast cancer cell lines and ERα-positive human breast cancer tissue was evaluated by immunofluorescence and immunohistochemical assays.

**Results:**

A complex of Smad3/4 mediates TGF-β inhibition of ERα-mediated estrogenic activity of gene transcription in breast cancer cells, and Smad4 is essential and sufficient for such repression. Either overexpression of Smad3 or inhibition of Smad4 leads to the "switch" of TGF-β from a repressor to an activator. Down-regulation and abnormal cellular distribution of Smad4 were associated with some ERα-positive infiltrating human breast carcinoma. There appears a dynamic change of Smad4 expression from benign breast ductal tissue to infiltrating ductal carcinoma.

**Conclusion:**

These results suggest that aberrant expression of Smad4 or disruption of Smad4 activity lead to the loss of TGF-β suppression of ERα transactivity in breast cancer cells.

## Background

Estrogens act as mitogens to promote cell proliferation in both normal breast tissue and breast carcinomas through their binding to estrogen receptors (ER). The ERα is a transcriptional activator and regulates gene transcription either by directly binding to the estrogen-responsive element (ERE) or by interacting with other transcription factors [1,2]. Gene amplification or overexpression of ERα was found in some breast cancer [3,4]. Approximately 70% of breast cancers are ERα positive and estrogen dependent. ERα has become an important prognostic marker and a therapeutic target in breast cancer [5,6].

In contrast to estrogens, which induce proliferation of breast cancer cells, transforming growth factor-β (TGF-β) inhibits the growth of human breast cancer cells in culture [7,8]. TGF-β is the prototypic inhibitor of cell cycle progression and appears to directly antagonize the effects of many different mitogenic growth factors. A well-characterized TGF-β signaling pathway is initiated by the association between TGF-β and its two cell surface receptors, resulting in the formation of the receptor heterocomplex and activation of the type I receptor, which in turn activates the cytoplasmic receptor regulated-Smad (R-Smad: Smad2 and Smad3) proteins via phosphorylation [9]. Phosphorylated R-Smad associates with Smad4. The resulting heteromeric Smad complexes then translocate into the nucleus, where they regulate gene transcription in collaboration with other factors. The importance of the TGF-β signaling pathway in cancer development is underscored by the presence of downregulation or inactivating mutations in genes encoding TGF-β receptors and Smads in human carcinomas [10-12].

While the role of TGF-β in breast cancer is ambiguous, as it was shown to display both tumor-suppressing and -enhancing effects, loss of responsiveness to TGF-β is believed to be a major factor in tumor formation [13-15]. Activation of TGF-β represents one of the physiological countermeasures that are invoked to protect transformed cells against ERα excessive mitogenic stimulation. Additionally, inhibition of some breast cancer cell growth by tamoxifen appears to be mediated by TGF-β signaling pathway [16]. Inhibition of Tβ RII expression abolished antiestrogen-dependent growth inhibition [17,18]. It has been shown that Smad2, Smad3 and Smad4 all have physical interactions with ERα and that Smad4 acts as a transcriptional co-repressor for ERα and inhibits tumor growth by inducing apoptosis in ERα-positive cells [19-22]. Although the regulated gene targets of Smads/ERα have not been identified, these findings imply that Smads-mediated cross-talk with the estrogen receptor plays an important role in development and/or progression of breast cancer.

In this study, we investigated how TGF-β regulates ERα-induced gene transcription and potential mechanisms of frequent TGF-β resistance in breast cancer. We demonstrated that Smad4 is essential for TGF-β-mediated inhibition of ERα estrogenic transcription activity. Either overexpression of Smad3 or inhibition of Smad4 expression switches TGF-β to an activator for ERα transactivation in breast cancer cells. In addition, we found that the expression of Smad4 was downregulated with increased cytoplasmic localization in ERα-positive human infiltrating breast cancer tissue.

## Methods

### Cell Culture, Transient Transfection and Reporter Assays

MCF-7 cells were purchased from the American Type Culture Collection and maintained according to the manufacturer's instructions. MDA-MB-231 and MDA-MB-468 cells were a gift from Dr. Joseph Messina (Department of Pathology, University of Alabama at Birmingham, Birmingham, AL). These two cell lines were incubated in Leibovitz's L-15 medium with 2 mM L-glutamine (ATCC) supplemented with antibiotics and 10% fetal bovine serum (Cellgro) at 37°C in 5% CO_2_. COS-1 cells were incubated in Dulbecco's modified Eagle's medium (Cellgro) supplemented with antibiotics and 10% fetal bovine serum at 37% in 5% CO_2_. The methods used for transient transfection and luciferase assay have been described in Wu L. et al. [21]. Similarly, 2× ERE-TATA reporter plasmid was used to examine the function of TGF-β/Smads on the estrogen response element. The breast cancer cell line (MCF-7, MDA-MB-231 or MDA-MB-468) cells were split and plated at 5 × 10^4 ^cells/24-well plate. The cells were starved with Dulbecco's modified Eagle's phenol red-free medium supplemented with 10% charcoal-stripped bovine serum (Cellgro) for 24 hours. Transient transfections were performed using LipofectAMINE plus reagent (Invitrogen) with 0.2 μg of luciferase reporter and 2-20 ng (2 ng for MCF-7 cells; 20 ng for MDA-MB-231/468 cells) of the hERα expression plasmid. The amount of co-transfected Smad expression plasmid is indicated in the figures and text. Aliquots of cells were treated with E_2_, 1 nM (Sigma), TGF-β1, 100 pM (R&D), or a combination of E_2 _+ TGF-β1. Sixteen hours after the treatment, luciferase activity was assayed in each cell line using the Dual-luciferase™ assay kit (Promega) according to the manufacturer's instructions.

### Plasmids

HA-tagged human ERα plasmid was cloned into a pCDNA3 vector as described in previous studies [21]. The expression vectors pFLAG-Smad2, pFLAG-Smad3 and pFLAG-Smad4 were gifts from Dr. Rik Derynck (University of California at San Francisco, San Francisco, CA). The 2× ERE-TATA reporter was a gift from Dr. Valerie Clark (Duke University Medical Center, Durham, NC).

### Semi Quantitative Real Time PCR

The target cDNA sequence was evaluated using Primer3 http://frodo.wi.mit.edu/primer3. The sequences of primers used in RT-PCR assays are listed as follows: β-actin upper primer, 5'-AGACTTCGAGCAGGAGCTGG-3'; β-actin lower primer, 5'-CGGATGTCAACGTCACACTT-3'; *PS2 *upper primer, 5'-TTGTGGTTTTCCTGGTGTCA-3'; *PS2 *lower primer, 5'-CCGAGCTCTGGGACTAATCA-3'; *C-MYC *upper primer, 5'-CTCCTGGCAAAAGGTCAGAG-3'; *C-MYC *lower primer, 5'-TCGGTTGTTGCTGATCTGTC-3'. Total RNA was extracted from each breast cancer cell line using STAT-60 (TEL-TEST Inc.). The detailed procedures for mRNA purification, reverse transcription and semi quantitative real time PCR have been described previously [21].

### Co-immunoprecipitation and Immunoblotting assays

To detect endogenous-endogenous, endogenous-exogenous or exogenous-exogenous protein complexes, breast cancer cells or COS-1 cells were transfected with expression plasmid(s) encoding the indicated proteins or left untransfected. Thirty-six hours post-transfection, aliquots of cells were incubated for 1 hr in the presence or absence of β-estradiol (E_2_) and/or TGF-β1. Cells were lysed with radioimmune precipitation assay buffer [21], precleared with purified mouse or rabbit IgG [23], and centrifuged at 13,000 × g for 30 mins. Supernatants were incubated with antibodies and protein A/G beads for 3 hr at 4°C on a rotating platform. Immunoprecipitates were separated by 7%-12% SDS-PAGE and transferred to PVDF membranes (BioRad). Immunoblotting was carried out as previously described [21]. The antibodies used for immunoprecipitation and immunoblotting are listed as follows: anti-FLAG M2 (F3165, Sigma), anti-HA (rabbit polyclonal, H9658, Sigma), anti-Smad2/3 [23], anti-Smad4 (sc-7154, and sc-7966, Santa Cruz), and anti-ERα (sc-8002, Santa Cruz).

### Immunofluorescence Detection

The procedure for immunofluorescence staining was adapted from the online protocol http://www.bdbiosciences.com/pharmingen/protocols/Immunofluorescence_Microscopy.shtml of BD Biosciences: Pharmingen: Protocols. The primary antibodies used were: monoclonal mouse anti-Smad2/3 (S66220, BD Transduction Laboratories) and polyclonal rabbit anti-Smad4 (sc-7154, Santa Cruz). The secondary Texas-red™-conjugated goat anti-rabbit and FITC-conjugated goat anti-mouse antibodies were used for immunofluorescence detection. The DNA dye 4',6-diamidino-2-phenylindole, dihydrochloride (DAPI) (D21490, "FluoroPureTM" grade, Molecular Probes) was used after the secondary antibodies to visualize the nucleus of the cells. Images were captured with an Olympus IX70 inverted fluorescence microscope and MagnaFire SP (Optronics) digital camera and visualized using Adobe Photoshop 7.0 and Image-pro^® ^Express 4.5 (MediaCybernetics Inc.) software.

### Immunohistochemical Assay

Immunohistochemical staining was performed on serial paraffin-embedded sections of human breast tissue using UltraVision One detection system (HRP polymer & DAB Plus chromogen, Thermo Fisher Scientific, Fremont, CA). The mouse monoclonal antibody against Smad4 was used at a dilution of 1:250. Sections of 4 μm were deparaffinized and boiled in Tris-EDTA buffer (10 mM Tris Base, 1 mM EDTA solution, 0.05% Tween 20, pH9.0) for 15 minutes. They were incubated with Ultra V Block for 5 min to block nonspecific binding. The sections were then incubated overnight with anti-Smad4 antibody at 4°C. After washing in TBS 0.025% Triton X-100, the slides were incubated in Hydrogen Peroxide Block for 10 minutes. After washing in TBS buffer, they were incubated with UltraVison One HRP Polymer (second antibodies) for 30 minutes, followed by staining with DAB Plus Chromogen and Substrate. Counterstaining was performed with hematoxylin.

### Sequential DNA Precipitation Assay

To study the ERα-associated ERE complex, COS-1 cells were co-transfected with HA-tagged ERα, FLAG-tagged Smad3 and FLAG-tagged Smad4. The ERα-containing protein complexes were first immunoprecipitated with an anti-HA antibody and eluted with HA-tagged peptide (I2149, Sigma). The eluates were then subjected to DNA precipitation assays as described by Chen et al [23] and Bonni et al [24]. The sequences of biotin-labeled wild-type and mutant ERE oligonucleotides are listed as follows: Biotin-ERE (forward): 5'-GATCTCGAGTCAGGTCACAGTGACCTGA-3'; Biotin-ERE (reverse): 5'-TCAGGTCACTGTGACCTGACTCGAGATC-3'; Biotin-mutantERE (forward): 5'-GATCTCGAGTCACCGCACAGTGAAATGA-3'; Biotin-mutantERE (reverse): 5'-TCATTTCACTGTGCGGTGACTCGAGATC-3'. Finally, the precipitated protein complexes which contain ERE-bound ERα were examined by immunoblotting assays with an anti-FLAG antibody.

### Small Interfering RNA (siRNA)

siRNA targeting Smad4 was prepared by cloning between the ApaI and EcoRI site of the Multi Cloning Sites of the pBS/U6 vector. The specific sequence for Smad4 was determined with assistance from siRNA Target Finder (Ambion^®^, Inc.). DNA oligonucleotides with the following sequences, along with their complementary strands, were synthesized by Operon (QIAGEN): siRNA for Smad4:

Forward: 5'-CATTGGATGGGAGGCTTCATTCAAGCTTTGAAGCCTCCCATCCAA TGTTTTTT-3'; Reverse: 5'-AATTAAAAAACATTGGATGGGAGGCTTCAAAGCTT GAATGAAGCCTCCCATCCAATGGGCC-3'. Similar oligonucleotides with the following scrambled sequences were designed as negative controls:

Forward: 5'-TAGTCTAGGAGGTCGAGTCTTCAAGCTTGACTGACCTCCTAGACT ATTTTTT-3'; Reverse: 5'-AATTAAAAAATAGTCTAGGAGGTCGAGTCAAGCTTG AAGACTCGACCTCCTAGACTAGGCC-3'. Each pair of DNA oligos was annealed and cloned into the pBS/U6 vector between ApaI and EcoRI site. MCF-7 cells were transfected with siRNA using LipofectAMINE plus reagent. Sixty hours after transfection, cells were treated with or without E_2 _and/or TGF-β for 8 hours. Total RNA and cell lysates were prepared and subjected to RT-PCR or western blotting analysis respectively.

## Results

### The Smad3/4 Complex Confers TGF-β Repression of ERα-Mediated Gene Transcription

**Figure 1:**
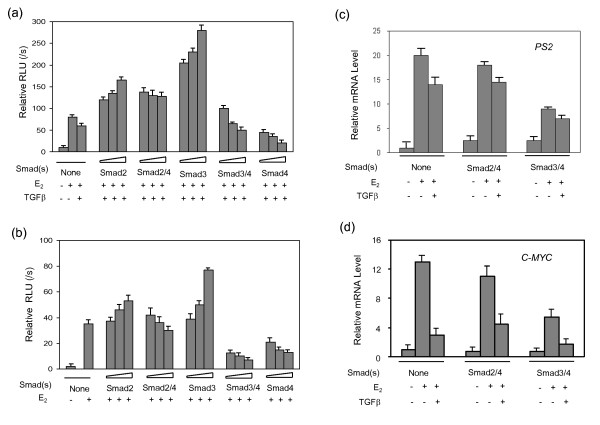
**The Smad3/4 complex confers TGF-β repression on ERα-mediated transcription**. Smads (Smad2, Smad3, and Smad4 alone or combinations of Smad2/4 or Smad3/4) or empty vector was co-transfected into MCF-7 cells (a) or MDA-MB-468 cells (b). The cells were treated with (+) or without (-) E_2 _and/or TGF-β as indicated for 16 hours prior to lysis, and analyzed for luciferase activity. (c-d) MCF-7 cells were transfected with Smad2/4 or Smad3/4 and treated with (+) or without (-) E2 and/or TGF-β as indicated for 8 hours. Total mRNA was isolated and the mRNA levels of *PS2 *and *C-MYC *were detected and quantified by reverse transcription and semi-quantitative real time PCR.

The complex of R-Smads and Smad4 forms the core of transcriptional regulatory machinery in TGF-β signaling. We have demonstrated that the expression of Smad4 without TGF-β treatment inhibited ERα-mediated transcription in a dose-dependent manner [21]. Both Smad3 and Smad2 were shown to interact with ERα, thereby having a direct impact on both TGF-β and estrogen signaling pathways [19,21,22]. We therefore examined how the complexes of Smad4 and R-Smads mediate estrogen signal transduction through their interactions with ERα in breast cancer cells. We first performed a reporter assay using a construct bearing two repeats of the estrogen response element (ERE) and luciferase cDNA (2× ERE-TATA-LUC). MCF-7 breast cancer cells were co-transfected with expression plasmids of Smad2, Smad3, Smad4, or Smad4 in combination with either Smad2 or Smad3. The cells were then treated for 16 hours with either β-estradiol (E_2_) alone or E_2 _in combination with TGF-β1. As shown in Figure 1a, expression of either Smad2 or Smad3 enhanced E_2_-induced luciferase transcription in a dose-dependent manner. Smad2 had a limited effect. Overexpression of Smad4 alone or with Smad3 strongly attenuated E_2_-induced luciferase activity, whereas Smad2/4 only showed moderate inhibition. These results clearly indicate that Smad3 and Smad4 act as ERα coactivator and corepressor, respectively, and that Smad4 overrides Smad3/4 in the repression of ERα-induced transcription. Apparently, Smad2 is not a potent ERα coactivator, nor is the Smad2/4 complex an effective repressor. To confirm this observation, we performed a similar experiment in the breast cancer cell line MDA-MB-468 which lacks endogenous ERα and Smad4 [25]. By transient expression of a small amount of ERα, we reconstituted ERα-mediated signaling. Overexpression of Smad3 enhanced ERα-induced transcription, while Smad4 alone or in complex with Smad3 inhibited ERα transcription activity (Figure 1b). These results are similar to those observed in MCF-7 cells.

ERα regulates gene transcription either by binding directly to the promoter of target genes (such as *PS2*) or by binding indirectly through a mechanism involving other transcription factors such as Sp1 and AP1 (such as *C-MYC*). To determine whether Smad4 and R-Smads also regulate ERα downstream genes, we measured the endogenous mRNA levels of *PS2 *and *C-MYC*, using endogenous β-actin mRNA as a control. The MCF-7 hormone-dependent breast cancer cell line was transiently transfected with Smad2/4 or Smad3/4 and treated with TGF-β1 for 8 hours. Total mRNA was extracted followed by reverse transcription and semi-quantitative real time PCR. As shown in Figures 1c and 1d, TGF-β downregulated the expression levels of *PS2 *and *C-MYC *mRNA stimulated by E_2_. Transient expression of Smad3/4 repressed the transcription of these two genes, while overexpression of Smad2/4 did not significantly alter the transcriptional activity. These results clearly indicate that, in breast cancer cell lines, Smad4 can either function as an ERα transcriptional co-repressor on its own or mediate TGF-β suppression by forming a complex with Smad3.

### Smad4 Is Essential For TGF-β-Mediated Repression of ERα Transactivation

**Figure 2:**
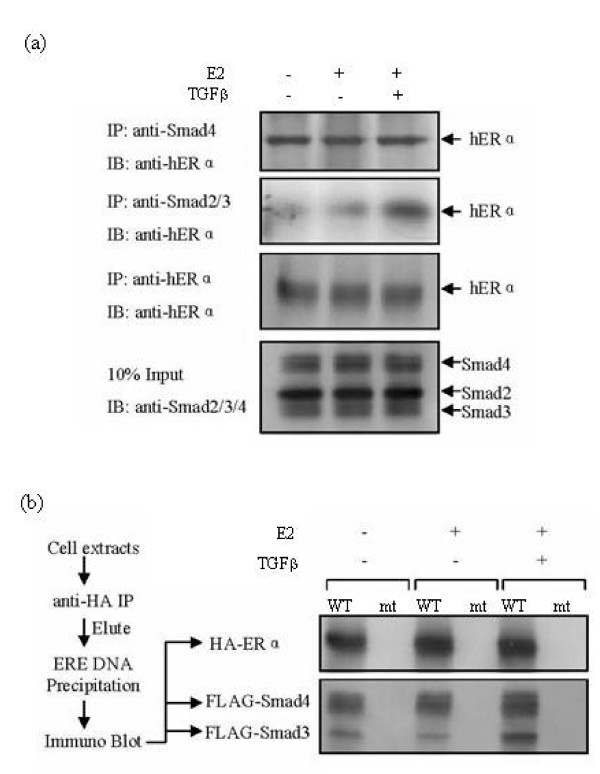
**The EREaccommodates Smad3/4 and ERα complexes in the nucleus**. (a) MCF-7 cells were treated with E_2_, E_2 _+ TGF-β1 or vehicle (as a negative control) for 1 hr as indicated. Lysates were subjected to immunoprecipitation with anti-Smad4, anti-Smad2/Smad3 or anti-ERα antibody. The Smad-associated endogenous ERα was detected by immunoblot analysis. The expression levels of endogenous proteins were monitored by immunoblotting of total cell protein. (b) COS-1 cells were transfected with HA-ERα, FLAG-Smad3 and FLAG-Smad4 expression plasmids. Cells were treated with (+) or without (-) E_2 _and TGF-β1 and subjected to sequential immunoprecipitation, DNA pull-down (DNAP), and immunoblotting. Wild-type (WT) and mutant (mt) biotinylated ERE oligonucleotides were used in the DNAP. The presence of HA-ERα, FLAG-Smad3 and FLAG-Smad4 in the DNAP was detected with anti-FLAG and anti-HA antibodies.

Our next objective was to investigate how Smad3 and Smad4 can act differently in regulating ERα transcriptional activity. Smad3 is known to form a complex with Smad4 and translocate into the nucleus upon TGF-β-induced phosphorylation. Similarly, the estrogen receptor, upon binding an estrogenic ligand such as E_2_, dimerizes and translocates into the nucleus. Therefore, we employed a co-immunoprecipitation (co-IP) assay to determine whether TGF-β and E_2 _can regulate the interaction of ERα with Smad3 and Smad4. MCF-7 breast cancer cells were treated with either E_2 _alone or E_2 _together with TGF-β1. The whole cell lysates were subjected to co-IP assays using anti-Smad4 or anti-Smad2/3 antibodies. The results demonstrated that the interaction between Smad2/3 and ERα was enhanced within 1 hr of TGF-β addition, while Smad4 binding ERα was not dependent upon either E_2 _or TGF-β1 stimulation (Figure 2a). Immunoprecipitation with a control mouse or rabbit IgG revealed no detected product (data not shown). The canonical transcriptional activation of ER target genes is attributable to the direct binding of ER to the estrogen responsive element in the promoter region of ER target genes. In our previous studies with chromatin immunoprecipitation assay [21], we demonstrated the Smad4 expression did not inhibit ERα binding to the chromatin. To further examine if Smad3/Smad4/ERα complex can bind to the ERE, we performed a sequential DNA precipitation assay [23]. FLAG-tagged Smad3 and Smad4 were co-transfected into COS-1 cells with HA-tagged ERα expression plasmids. The cells were then treated with E_2_, TGF-β or E_2 _+ TGF-β. HA-tagged ERα was precipitated from cell extracts with anti-HA antibody. The precipitates were eluted with an HA peptide and subjected to DNA precipitation (DNAP) with either biotinylated ERE double-stranded DNA oligonucleotides (wild-type) or a disrupted sequence (mutant) as a control for the experiment. The precipitated protein complexes were examined by anti-FLAG, anti-HA, and control IgG antibodies. As shown in Figure 2b, the wild-type ERE probe precipitated ERα, Smad4 and a noticeable amount of Smad3. Immunoblotting with a control IgG revealed no detected bands. A treatment with TGF-β1 enhanced the association of Smad3, but not that of Smad4, with the ERα-containing ERE precipitates (Figure 2b), that are consistent with the co-IP assay results (Figure 2a). These data suggest that Smad4 can function as an ERα corepressor independent of TGF-β signaling, whereas Smad3 acts as a TGF-β-regulated cofactor for ERα.

**Figure 3:**
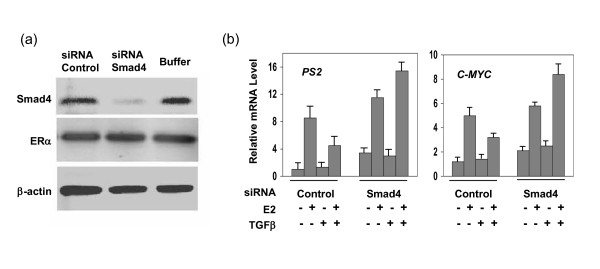
**Smad4 is essential for the inhibitory effect of TGF-β in ERα-mediated transcriptional activation**. (a) RNAi was performed in MCF-7 cells using Smad4 siRNA (see materials and methods). (b) MCF-7 cells were transfected with either siRNA targeting Smad4 or scrambled siRNA (as a control). Sixty hours after transfection, the cells were treated with (+) or without (-) E_2 _or TGF-β for 6 hrs. Total RNA was isolated and the mRNA levels of *PS2 *and *C-MYC *were quantitated by RT-PCR.

We previously demonstrated that Smad4 through its MH1 domain and linker region interacted with the activation function 1 domain of ERα and that the ectopic expression of Smad4 inhibited E2- induced luciferase activity in a dose-dependent manner [21]. In addition, in breast T47D cells with undetected Smad4, ectopic expression of Smad4 resulted in inhibition of endogenous ERα direct response genes [21]. To further examine the essential role of Smad4 in the cross-talk between TGF-β and estrogen signaling, we reduced the expression level of endogenous Smad4 using a small interfering RNA (siRNA) gene silencing technique. As shown in Figure 3a, Smad4 protein was almost non-detectable in the MCF-7 cells treated with Smad4-specific siRNA (siRNA-Smad4), while treatment of the MCF-7 cells with a non-specific siRNA (siRNA-Control) did not change the protein level of Smad4 or other control genes. The effect of TGF-β on ERα-induced transcription was measured on two ERα downstream genes *PS2 *and *C-MYC*. As expected, transcription of both genes in the control cells (treated with siRNA-Control) was up-regulated by E_2 _treatment and down-regulated by TGF-β (Figure 3b). In the siRNA-Smad4 treated cells, however, E_2 _induction enhanced the expression of both *PS2 *and *C-MYC *genes. Moreover, the addition of TGF-β did not result in repression but instead stimulated estrogen-induced *PS2 *and *C-MYC *transcription (Figure 3b). Our findings indicate that the reduction of Smad4 protein attenuates the inhibitory effect of TGF-β on estrogen-induced gene transcription. In the absence of Smad4, TGF-β can regulate ERα signaling through Smad3 and thereby might be converted from a repressor to an activator of certain ER target gene responses.

### Abnormal Expression of Smad4 is Detected Some ERα-positive Human Infiltrating Breast Carcinoma

**Figure 4:**
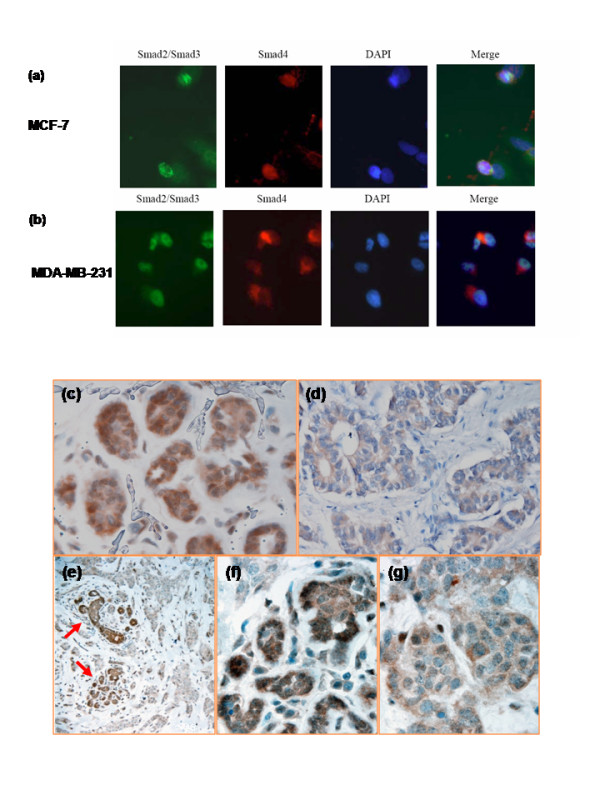
**Expression of Smad4 in breast cancer cell lines and ERα-positive infiltrating beast carcinoma**. (a and b) MCF-7 and MDA-MB-231 cells were treated with TGF-β for 1 hr and then subjected to immunofluorescence analysis with anti-Smad2/3 and anti-Smad4 antibodies to localize endogenous Smad2/3 and Smad4. Nuclei were visualized by DNA staining with DAPI. (c-g) Immunohistochemical staining of Smad4 in human breast tissue: (c) Benign breast ductal epithelial (1000×). (d) ERα-positive infiltrating breast carcinoma (1000×). (e) Residual benign ductal epithelial (arrow) and surrounding ERα-positive infiltrating breast carcinoma (200×). (f) Residual benign breast ductal epithelial in panel e (1000×). (g) Surrounding infiltrating breast carcinoma in panel e (1000×).

Tumor cells often escape TGF-β growth inhibition through the loss of key signaling transducers in the pathway. Our findings suggest that Smad4 is required for TGF-β to inhibit ERα-induced transcription, which provides important information regarding TGF-β resistance in breast cancer cells. It has been reported that altered expression levels or/and abnormal cellular distribution of biologically active proteins are associated with some human neoplasm [26-29]. We set to assess smad4 expression in human breast cancer cell lines and breast cancer tissue. While most breast cancer cell lines (e.g., MDA-MB-231) are essentially refractory to the growth inhibition, the MCF-7 cell line responds to TGF-β by suppression of cell growth [30,31]. We employed both MCF-7 and MDA-MB-231 to determine whether there is any difference in the expression of Smad4 protein. TGF-β stimulated translocation of Smad2/3 with Smad4 into the nucleus of MCF-7 cells (Figure 4a). In the MDA-MB-231 cells, however, Smad4 did not translocate into the nucleus with Smad2/3 in response to TGF-β stimulation (Figure 4b). These data suggest that cytoplamic retaining of Smad4 upon TGF-β stimulation contributes to the resistance of MDA-MB-231 to TGF-β growth inhibition. We then examined Smad4 expression in benign human breast and ERα-positive infiltrating ductal carcinoma tissue. Smad4 was strongly expressed in both cytoplasm and nucleus of benign breast ductal epithelial cells from 4 individuals (Figure 4c). In contrast, Smad4 are weekly expressed and largely restricted in the cytoplasm of cancer cells from 8 of 12 cases with infiltrating ductal carcinoma (Figure 4d). The variable cytoplasmic and nuclear expression of Smad4 was detected in the remaining 4 cases. To further investigate the significance of Smad4 expression in breast cancer, we examined the specimen with both benign ductal epithelial and cancer cells. As shown in Figure 4e-g, the residual benign ductal epithelial cells surrounded by infiltrating ductal carcinoma highly expressed Smad4 in both cytoplasm and nucleus, while the expression of Smad4 was markedly decreased and largely restricted to cytoplasm in ERα-positive infiltrating ductal carcinoma. These results demonstrate that the reduced expression and abnormal cytoplasmic retaining of Smad4 protein are present in some of ERα-positive infiltrating breast ductal carcinoma. The data also clearly indicate a dynamic change of Smad4 expression from benign breast ductal tissue to infiltrating ductal carcinoma.

## Discussion

Previous investigations have demonstrated that ER status is a very important factor in the management of breast cancer, and that suppression of ER mitogenic activity is a viable strategy for treatment and prevention of breast cancer. TGF-β is a natural negative growth regulator of epithelial cells during both development and tumorigenesis of the mammary gland. Most breast carcinomas are refractory to the suppressive effect of TGF-β with elevated TGF-β expression, suggesting an important role of TGF-β in breast cancer tumorigenesis.

In previous studies, we have demonstrated that Smad4 can act as a transcriptional corepressor for ERα [21]. Smad3, on the other hand, has been shown to act as a coactivator for ERα [19]. Since TGF-β activation leads to the formation of an active Smad3/4 complex which directly binds to the promoter of many TGF-β responsive genes, it is conceivable that the Smad3/4 complex, rather than Smad3 or Smad4 alone, plays a major role in the crosstalk between TGF-β and estrogen. Our data clearly indicate that the Smad3/4 complex plays the same role as Smad4 alone, i.e., inhibiting ERα-mediated gene transcription. This observation is not surprising since TGF-β treatment alone suppresses estrogen-induced ER target gene expression. However, it adds to the complexity of the question why Smad3 and Smad4 act in opposite ways in the regulation of estrogen signaling, while they have the same effect on TGF-β signaling. One possible explanation is that, in the context of ERα-mediated transcription, Smad3 and Smad4 *per se *may recruit functionally different cofactors such as histone acetylases and histone deacetylases. The protein complex(es) recruited by Smad3 or Smad4 might determine the role of Smad3 or Smad4 as a transcriptional cofactor in estrogen signaling. In breast epithelial cells that have intact TGF-β/Smad and estrogen signaling networks, the "net effect" of TGF-β may be determined by the protein complex that incorporated both Smad3 and Smad4. In breast carcinoma cells that lack one component of the Smad3/4 complex, Smad3 or Smad4 may act as a coactivator or corepressor for ERα that confers a TGF-β activating or suppressing signal on the estrogen signaling pathway. The high frequency of Smad4 mutations in human tumors [32-35] suggests a role for Smad4 as a tumor suppressor independent of TGF-β signaling. Smad4 mutations in breast carcinoma have also been reported [36,37]. In addition, the work on Smad4 conditional knockout mice also indicates that Smad4 is required for the suppression function of TGF-β in the proliferation of mammary epithelial cells [38].

We have shown that, in the presence of Smad4, TGF-β inhibits ERα-mediated transcriptional activity. A decrease in Smad4 expression partially or completely abrogates TGF-β suppression of ERα-target genes, and TGF-β can further activate ER-target genes since Smad3 acts as a transcriptional coactivator for ERα (Figure 1a). It seems that TGF-β can regulate ERα estrogenic transcriptional activity in both a negative and positive manner, and apparently, that the absence of Smad4 can switch TGF-β from a suppressor to an activator of ER signaling. TGF-β dual regulation of ERα transcriptional activity may also explain the biphasic effect in tumors: suppression of tumor growth at the early stages and promotion of tumor spreading during the later stages. Further investigations are needed to identify the target genes that are specifically regulated by R-Smads and/or Smad4, especially in the context of tumor cells. Hopefully, the results from future studies will lead to a better understanding of the dual role of TGF-β in cancer development.

Loss of TGF-β inhibition can be due to mutations or deletions of TGF-β signaling components. Indeed, mutations of TGF-β receptor or Smads-encoding genes have been reported in different forms of cancers, such as colon cancer and pancreatic cancer. Abnormal expression level and cellular distribution of biologically active proteins have been implicated in the tumorigenesis. Aberrant cytoplasmic localization of nucleophosmin in primary acute myeloid leukemia has been implicated in disrupting ARF-MDM2-p53 signal pathway and contributed to leukemogenesis [39,40]. Significant reduced expression of Smad4 protein was found in some human carcinoma including breast cancer [41-44]. Our data demonstrate that, in TGF-β-treated MDA-MB-231 cells, Smad4 failed to translocate into the nucleus with Smad2/3, therefore losing the function of Smad4 as a repressor in the nucleus and probably contributing to decreased growth inhibition of TGF-β in this cell line. In addition, the expression of Smad4 are decreased and largely restricted in the cytoplasm of some of ERα-positive infiltrating human breast cancer cells in contrast to the benign breast tissue in which Smad4 was strongly expressed in both cytoplasm and nucleus of ductal epithelial cells. Moreover, the marked difference of Smad4 expression is noted between carcinoma cells and surrounding residual normal ductal tissue from same specimen (Fig. 4e-g). These data indicate that the dysregulation of Smad4 protein expression may play a role in the development and progression of ERα-positive breast carcinoma. The fact that aberrant expression of Smad4 is only seen some of ERα-positive infiltrating breast cancer is consistent with the heterogeneity of human breast carcinoma in biological features, development and progression, and therapy response. It is likely that in an ERα-positive breast carcinoma with aberrant expression of Smad4, TGF-β not only is unable to inhibit tumor growth, but also promote tumor progression through enhancing the estrogen-ERα mediated cell proliferation. The mechanism for dysregulation of Smad4 expression in ERα-positive infiltrating breast cancer is still unknown. It could be due to mutations or deletion of Smad4, or aberrant expression of certain oncoproteins. Our findings may provide a basic mechanism to explain the lack of TGF-β responsiveness and anti-estrogen-dependent growth inhibition in some of ERα-positive infiltrating breast carcinoma, support an idea of both tumor suppressing and enhancing effects of TGF-β in the development and/or progression of breast cancer, and open a path to further investigate the cause of aberrant expression of Smad4 in ERα-positive infiltrating breast carcinoma.

## Conclusion

We have shown that TGF-β can regulate ERα estrogenic transcriptional activity in both negative and positive manners. Smad4 is essential for TGF-β-mediated inhibition of ERα estrogenic transcription activity, and the inhibition of Smad4 expression switches TGF-β from a repressor to an activator for ERα transactivation in breast cancer cells. We have demonstrated that down-regulation and relatively increased cytoplasmic localization of Smad4 were associated with some ERα-positive infiltrating human breast carcinoma. These results suggest that aberrant expression of Smad4 or disruption of Smad4 activity be one of mechanisms for loss of TGF-β negative regulation on ERα transcriptional activity in breast cancer.

## Competing interests

The authors declare that they have no competing interests.

## Authors' contributions

YR and LW carried out the molecular and cell biology and immunohistochemistry studies, and wrote the manuscript. ARF participated in the immunofluorescence microscopy studies. WG participated in data analysis. XC and WM designed the research, analyzed the data, and revised the manuscript. All authors read and approved the final manuscript.
